# Examination of the skeletal proteome of the brittle star *Ophiocoma* wendtii reveals overall conservation of proteins but variation in spicule matrix proteins

**DOI:** 10.1186/s12953-015-0064-7

**Published:** 2015-02-07

**Authors:** Ryan W Seaver, Brian T Livingston

**Affiliations:** Department of Biological Sciences, California State University, Long Beach, 1250 Bellflower Blvd, 90840 Long Beach, CA USA

**Keywords:** Skeletal proteome, Echinoderms

## Abstract

**Background:**

While formation of mineralized tissue is characteristic of many animal taxa, the proteins that interact with mineral are diverse and appear in many cases to be of independent origin. Extracellular matrix proteins involved in mineralization do share some common features. They tend to be disordered, secreted proteins with repetitive, low complexity. The genes encoding these proteins are often duplicated and undergo concerted evolution, further diversifying the repetitive domains. This makes it difficult to identify mineralization genes and the proteins they encode using bioinformatics techniques. Here we describe the use of proteomics to identify mineralization genes in an ophiuroid echinoderm, *Ophiocoma wendtii* (*O. wendtii*).

**Results:**

We have isolated the occluded proteins within the mineralized tissue of the brittle star *Ophiocoma wendtii*. The proteins were analyzed both unfractionated and separated on SDS-PAGE gels. Each slice was analyzed using mass spectroscopy and the amino acid sequence of the most prevalent peptides was obtained. This was compared to both an embryonic transcriptome from the gastrula stage when skeleton is being formed and a tube foot (an adult mineralized tissue) transcriptome. Thirty eight proteins were identified which matched known proteins or protein domains in the NCBI databases. These include C-type lectins, ECM proteins, Kazal-type protease inhibitors, matrix metalloproteases as well as more common cellular proteins. Many of these are similar to those found in the sea urchin *Strongylocentrotus purpuratus* (S. purpuratus) skeleton. We did not, however, identify clear homologs to the sea urchin spicule matrix proteins, and the number of C-type lectin containing genes was much reduced compared to sea urchins. Also notably absent was MSP-130.

**Conclusions:**

Our results show an overall conservation of the types of proteins found in the mineralized tissues of two divergent groups of echinoderms, as well as in mineralized tissues in general. However, the extensive gene duplication and concerted evolution seen in the spicule matrix proteins found in the sea urchin skeleton was not observed in the brittle star.

**Electronic supplementary material:**

The online version of this article (doi:10.1186/s12953-015-0064-7) contains supplementary material, which is available to authorized users.

## Background

The development of mineralized tissues has played a significant role in the evolution of animals. The rapid appearance of such structures over a relatively short time period during the Cambrian explosion of animal forms seen in the fossil record is indicative of the selective advantage mineralized structures conferred upon the animals that possessed them. Mineralized structures appeared across all taxa and are currently seen in some form in most extant groups [1]. Studies on both vertebrates [2-4] and mollusks [5,6]suggest that the evolution of the proteins involved in mineralization occurred independently several times, but that it is convergent in that the proteins in different groups share features. In all groups that have been studied mineral is deposited onto a matrix of secreted proteins. Many of these proteins are flexible, acidic glycoproteins, often with lectin binding domains and repetitive low complexity domains. However, the overall characteristics of the proteins rather than the primary sequence seems to be what is selected for, making standard bioinformatics approaches to identify and compare homologous genes and proteins from different organisms ineffective [7]. The lack of conserved domains has complicated our understanding of the mechanisms by which these proteins mediate the process of biomineralization.

Skeleton formation in the sea urchin *Strongylocentrotus purpuratus* has been particularly well studied. The genome for this species has been sequenced and annotated [8], facilitating the identification of the proteome. Skeletal elements can be isolated from both adult and embryonic tissues and all cellular material removed. The clean skeletal elements can then be demineralized, leaving behind the occluded proteins that make up the protein matrix that forms the scaffold for mineral deposition [9]. Using these methods, both the adult and embryonic skeletal proteomes have been determined for the sea urchin *S. purpuratus* [10-12]. The *S. purpuratus* skeletal proteome contains a family of proteins that contain C-type lectin domains, are acidic, and contain repetitive regions of similar amino acid content, but without identical conserved sequences. These proteins appear to have arisen through gene duplication and concerted evolution within the repetitive domains [13]. We have examined the evolution of the gene that encodes SM50, one of the spicule matrix proteins isolated from *S. purpuratus*, in different species of sea urchins [7,14]. While the sequence of the C-type lectin domain has been relatively conserved, the repetitive domain has diverged both in repeat number and sequence between species, and even within a species. SM50 can be found in all euechinoids, but, it has not been found in the related cidaroid urchins.

All echinoderms form mineralized structures in the adult, and Ophiuroids, or brittle stars, and Echinoids, or sea urchins, form an extensive skeleton in the embryo and larval stages. Sea urchins and Holothuroids (sea cucumbers) are sister taxa, but the cucumbers do not form extensive larval skeletons, and have much reduced adult skeleton. Asteroids (sea stars) do not form a larval skeleton at all. Because of this, we wanted to determine if the proteins involved in skeleton formation in brittle stars were similar to that of sea urchins, suggesting a common origin with a subsequent loss in holothuroids, or whether the skeletons are formed by novel molecular processes in both species, suggesting convergent evolution. We have characterized the transcriptome of the brittle star *Ophiocoma wendtii* [15] at the gastrula stage of embryonic development when a skeleton is being formed. We were not able to identify homologues of the sea urchin spicule matrix genes expressed in the brittle star at this stage. We did identify some transcripts that encoded proteins similar to that found in the sea urchin proteome, but not with enough certainty to claim they participate in skeleton formation.

Here we report the mass spectrometry analysis of the proteins occluded in the *Ophiocoma wendtii* skeleton and comparison of this data to both the gastrula transcriptome and to a transcriptome isolated from an adult mineralized tissue, the tube foot. We have identified thirty eight proteins with at least some similarity to previously identified proteins. The resulting proteome is compared to that of the sea urchin *Strongylocentrotus purpuratus*.

## Results and discussion

**Figure 1:**
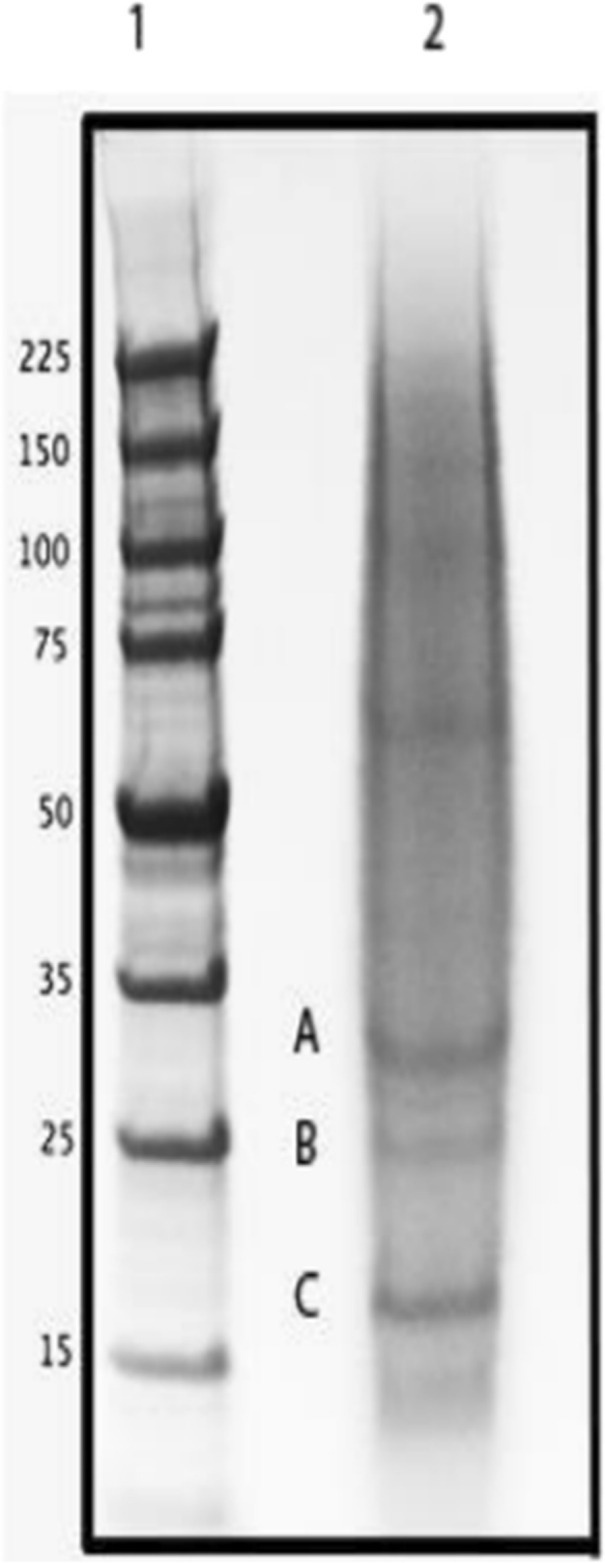
**SDS-PAGE gel of** ***O. wendtii*** **proteins isolated from the adult skeleton. 1:** MW markers, **2:** *O. wendtii*protein. **A, B and C** indicate major protein bands excised for LC/MS/MS analysis.

Skeletal protein separated by SDS-PAGE and fractionated into either 20 equal slices or into the three major bands (Figure 1). Following digestion and LC-MS/MS, the peptide sequences were compared against transcriptome sequence databases from the gastrula stage of embryonic development and from adult tube feet. Both of these samples represent tissues that are actively synthesizing skeleton. In the sea urchin, the majority of skeletogenic proteins are expressed in both the adult and the embryo [10-12]. The transcriptome sequences do not usually contain full length coding regions, but the coverage of the transcriptomes was high [15] and utilization of two databases enhances protein identification. In the 20-band sample, a total of 3721 spectra yielded 408 unique peptides (Additional file 1). Peptides mapped to regions of transcripts with stop codons or that were preceded by a stop codon without an intervening start codon were removed. Several peptides had ambiguous matches to multiple proteins, but in each case these proteins represented different transcripts in the same protein family that had common peptides. After pooling data sets, subtracting common contaminants, limiting proteins for quality, and removing spurious identifications, 37 positive hits to the brittle star gastrula transcriptome database were determined to be convincing. These correspond to sequenced transcripts that are expressed during the gastrula stage of development in *O. wendtii*, and the proteins they encode are enumerated in Table 1. The gastrula proteins were compared to *S. purpuratus* genome sequences and the S. purpuratus skeletal proteomes previously characterized [10-12]. 27 of these matched sequences in the NCBI databases (E- value cutoff of 10^−3^) and all 27 of the O. wendtii proteins had similarity to previously characterized *S. purpuratus* proteins or protein domains. Of the 27 proteins shared with *S. purpuratus*, 26 (96%) are found in S. purpuratus skeletal proteomes. Three of the *O. wendtii* proteins encode C-type lectins, a protein domain found in the abundant spicule matrix proteins in the S. purpuratus skeletal proteomes [10-12]. Also identified by comparison to NCBI and the *S. purpuratus* proteomes were a fibrinogen C/ficolin protein, an extracellular matrix [ECM] component,a matrix metalloproteinase [MMP] and two proteins with Kazal-type inhibitor domains. MMPs and proteins with Kazal inhibitor domains have been implicated in biomineralization in other organisms [3,16,17]. We also found two other proteins with calcium binding sites, calmodulin and an LDL receptor-like protein.

**Table 1:** **Proteomic identification of brittle star matrix proteins in gastrula**

***Ophiocoma wendtii*** **ID**	**Domains identified**, **possible homology**	**NCBI entry** ** [Sea Urchin] top hits**	**Presence in sea urchin skeletal proteome**	**Designation**
**Extracellular matrix components**				
LUPK	C-Type Lectin	XP_003730279.1	C-Type lectins found in all	SM22
266	C-Type Lectin	XP_003730279.1	C-Type lectins found in all	SM24
1464	C-Type Lectin	XP_790287.2	C-Type lectins found in all	SM21
358	Syndecan/SEA domain	NP_00118233.1	Spicule	
PO24	Fibrinogen C	XP_781712.2	Spicule and tooth	
**Protease and protease inhibitor domains**				
998	Agrin; Kazal Inhibitor	XP_791472.3	Test and tooth	
2557	Agrin; Kazal Inhibitor	XP_003724092.1	Test and Tooth	
NAKRO	Matrix Metalloproteinase	XP_791616.2	MMP's found in all	
2581	Alpha-2-macroglobulin	XP_788246.3	Found in all	
**Other proteins identified**				
149	Calmodulin	XP_780862.2	Test and Tooth	
M7PKJ	LDL receptor-like	XP_794505.3	Found in all	
LI454	LDL receptor-like	XM_003729261.1	Found in all	
8435	Defense protein 3-like	XP_789043.2	Not present	
KGGQ	Tetraspanin-like	XP_003726229.1	Spicule and Tooth	
FEQQ	LVN-1-like	XM_003723550.1	Not present	
12396	Actin	XP_001176242.1	Found in all	
144	Actin	NP_999634.1	Found in all	
14123	Actin	P53472.1	Found in all	
1QFC/3S4M	Collagen	NP_999675.1	Found in all	
7694	Glut peptide cyclotransferase	XP_003726229.1	Spicule and Tooth	
10842	Tubulin	XP_791790.1	Test and Spine	
83/1103/2063/4032	Ubiquitin	P23398.2	Found in all	

The unfractionated total protein sample isolated from skeleton was directly digested with trypsin and analyzed in order to identify the most prevalent proteins. The total protein solution yielded 25 spectra that identified 8 proteins with 90% confidence when compared to the gastrula transcriptome (Additional file 2). A C-type lectin domain containing protein (0266, Table 1) and a syndecan/SEA domain containing protein (358, Table 1) matched the majority of the spectra obtained. The other proteins identified had no conserved domains. We also identified a protein with a single peptide match that contained a C-type lectin domain, LUPK (Table 1). The identified cDNA sequence encoding LUPK was very short, so we obtained the full length sequence using RACE PCR and ran that against the LC/MS/MS data. This resulted in multiple peptide matches and a 100% protein identification score (Additional file 3). We also analyzed peptides generated by tryptic cleavage of proteins eluted from the three major bands identified on an SDS-PAGE gel (Figure 1). We identified several proteins listed in Table 1: an Agrin/Kazal inhibitor domain containing protein (998), Tetraspanin (KGGQ), Actin (144), C lectin SM24 (266), Syndecan/SEA domain containing protein (358) and Alpha-2 macroglobulin (2581). The number of peptide counts and percent of total spectra suggest that the SM24, Syndecan/SEA and actin proteins are most prevalent in these samples (Additional file 4).

The same spectra obtained from the 20 gel slices compared to the tube foot transcriptome database allowed the identification of 40 proteins with at least two peptide matches and a 95% protein probability. Of these, 25 matched known proteins in NCBI databases (Table 2), while 13 did not (Additional file 5). 2 proteins had no conserved domains, but matched genes in the S. purpuratus genome (Table 2). Of the 25 proteins identified, 23 were similar to proteins or protein domains found in the S. purpuratus skeletal proteomes (92%). 14 were shared with the proteins found in the gastrula proteome. Included in the tube foot proteins were all of the C-type lectins found using the gastrula transcriptome. An additional C-type lectin was found in the tube foot transcriptome, as well as a fibrinogen C-like protein and an additional ECM protein. The LDL receptor-like protein was also found, as well as several putative ion channels.

**Table 2:** **Proteomic identification of brittle star skeletal proteins in tube foot**

***Ophiocoma wendtii*** **ID**	**Domains identified, possible homology**	**NCBI entry [Sea Urchin] top hits**	**Presence in sea urchin skeletal proteome**	**Ophiocoma wendtii**	**Designation**
				**gastrula ID**	
**Extracellular matrix components**					
50743	C-Type Lectin	XP_003730279.1	C-Type lectins found in all	LUPK	SM22
7123	C-Type Lectin	XP_003730279.1	C-Type lectins found in all	266	SM24
40110	C-Type Lectin	XP_790287.2	C-Type lectins found in all	1464	SM21
4220	C-Type Lectin	NP999836.1	C-Type lectins found in all	none	SM20
40467	ECM-3 like	XP_003724641.1	Found in all	none	
67843/74501	Fibrinogen C	XP_783504.2	Spicule and tooth	TF only, similar to PO24	
**Protease domains**					
5998	Matrix Metalloproteinase	XP_791616.2	MMP's found in all	TF only, similar to NAKRO	
**Other proteins identified**					
2696	LDL receptor-like	XP_780169.2	Found in all	TF only, similar to M7PKJ	
6749	SCP domain	XP_781255	Similar domains present	TF only, similar to 2581	
32153	Gelsolin-like	XP_788777.2	Spine and tooth	none	
68329	Semaphorin 6D	XP_003729425.1	Spicule, test and tooth	none	
7643	Anion Exchange Pump	XP_791964.2	Spine and Tooth	none	
3983	Actin	NP_999634.1	Found in all	Homology unclear	
9834	Actin	1101351C	Found in all	Homology unclear	
5596	Beta-Tubulin	XP_791790.1	Test and Spine	TF only, similar to 842	
2896	Collagen	XP_795694.2	Found in all	Homology unclear	
9902	Collagen	NP_999675.1	Found in all	Homology unclear	
3947	Collagen	NP_999675.1	Found in all	Homology unclear	
453	Myosin	XP_785810.2	Tooth	Homology unclear	
7471	Ubiquitin	P23398.2	Found in all	Homology unclear	
3703	Ig Domain	XP_003723404.1	?	?	
4964	Cation channel?	XP_003727536.1	?	?	
9877	Cation channel?	XP_784612.2	?	?	
3	Unknown	XP_003729030	?	?	
2030	Unknown	XP_794671.2	?	?	

**Figure 2:**
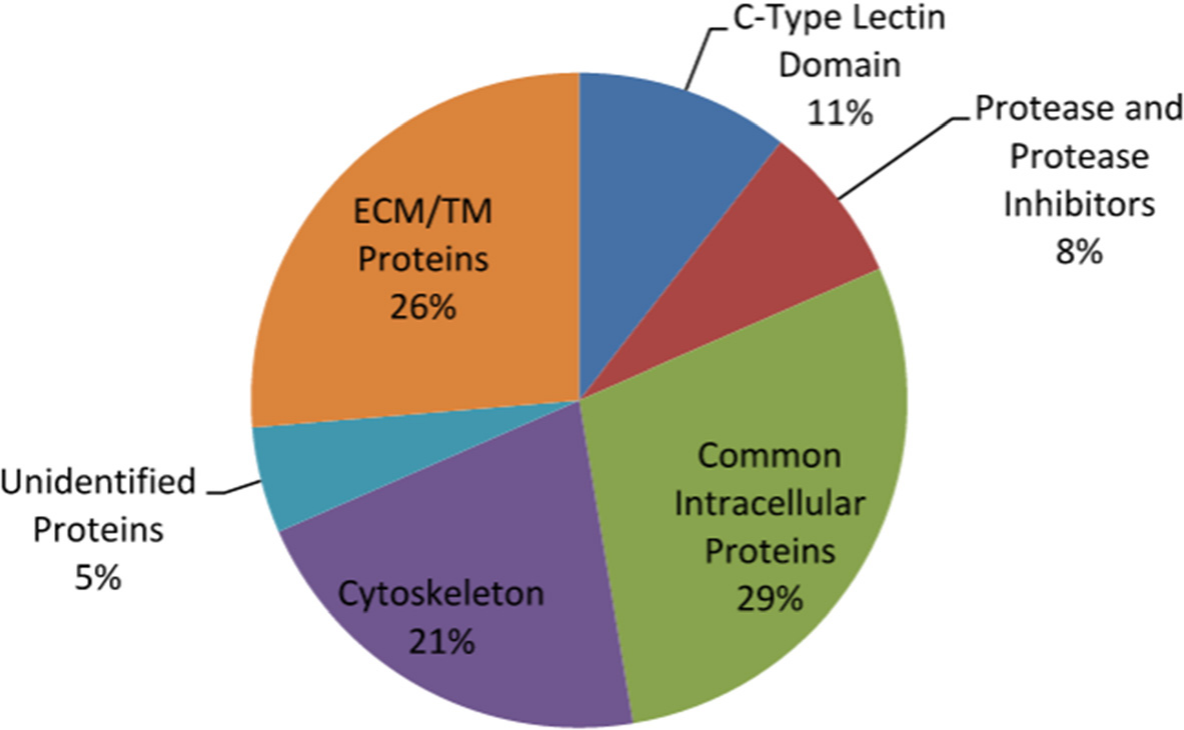
**Distribution of identified proteins among selected types of proteins.**

Of the 38 proteins that shared sequence similarity with previously identified proteins, 26% were ECM or transmembrane proteins, 11% were C-type lectins and 8% were proteases or protease inhibitors (Figure 2). These all represent functional classes of proteins that have been implicated in biomineralization. A large percentage of the proteins found represent functional classes that have not previously been thought to be critical in biomineralization (50%). However, many of the same proteins are also found in the S. purpuratus skeletal proteome (Tables 1 and 2). Although the data only provide an approximation of the abundance of these proteins, we feel we have identified the most prevalent protein components of the *O. wendtii* skeleton. The number of proteins we identified is much fewer than what is found in the four *S. purpuratus* skeletal proteomes characterized (10–12). This is likely due to the use of transcriptome databases rather than a complete genome. Still, this partial proteome allows us an opportunity to begin a comparative analysis of the proteins involved in biomineralization in these two echinoderm groups. There are still a number of proteins that did not match proteins of known function (see Additional files 1, 2, 4, 5) and some may play a role in mineralization. Of these, there is a set of predicted short proteins with long stretches of methionines identified. It is not known if these represent functional molecules. Since highly repetitive proteins are often found in biomineral (1,2,5,7,13), these may be of note.

### Proteins with C-type lectin domains

We identified four proteins containing C-type lectin domains. C-type lectin domain containing proteins have an important role for biomineralization in a range of species, but especially in echinoderms [13,18,19]. The level of conservation of C-type lectin domain containing proteins involved in biomineralization between echinoderm classes is unknown, so identifying this class of brittle star proteins that are involved in biomineralization is of special interest. Three of these also had low complexity domains (Table 3). These same three were also enriched in proline as well as amino acids found in the repetitive regions of the sea urchin spicule matrix proteins [7,14]. The pKA of these proteins is calculated to be acidic as well (Table 3). All of these characteristics are similar to what is seen in sea urchin spicule matrix proteins and proteins implicated in mineralization in other systems [2-5,10-12]. We designate these proteins OwSM20, OwSM21, OwSM22 and OwSM24 based on calculated molecular weights (Table 3). All of these proteins also have an amino terminal hydrophobic amino acid rich region that is likely functioning as a signal peptide, marking the cell for secretion to the matrix. This characteristic was also prevalent in sea urchin skeletal matrix proteins [13].

**Table 3:** **Characteristics of C lectin proteins**

**Identifier**	**Domain architecture**	**MW**	**pKA**	**Enriched amino acids**	**Designation**
266	C-type Lectin Domain; low complexity	24,192	4.46	P,Q,R,E rich	Ow SM24
1464	C-Type Lectin Domain; low complexity	21,463	5.33	P,Q,A,E rich	Ow SM21
LUPK	C-type lectin; low complexity	22,468	4.55	P rich	Ow SM22
4220	C-type Lectin Domain	20,522	4.86	None	Ow SM20

**Figure 3:**
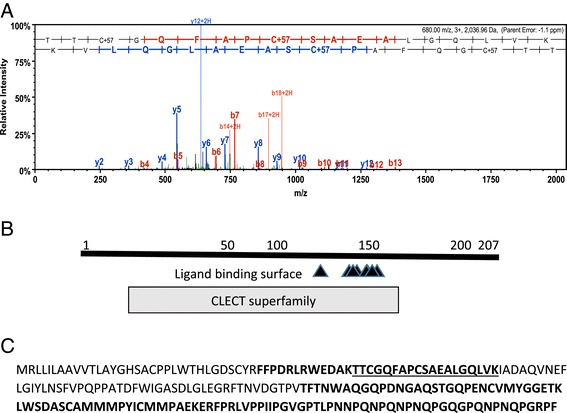
**Identification of a 24 kd skeletal protein with a C-type lectin domain. A. **Example spectra identifying Contig 266 as encoding a protein occluded in the O. wendtii skeleton. The predicted domain structure is shown in **B. C: **The open reading frame of Contig 266 with amino acid sequences identified using LC/MS/MS in bold. The amino acids identified in the spectrum in **A** are underlined.

**Figure 4:**
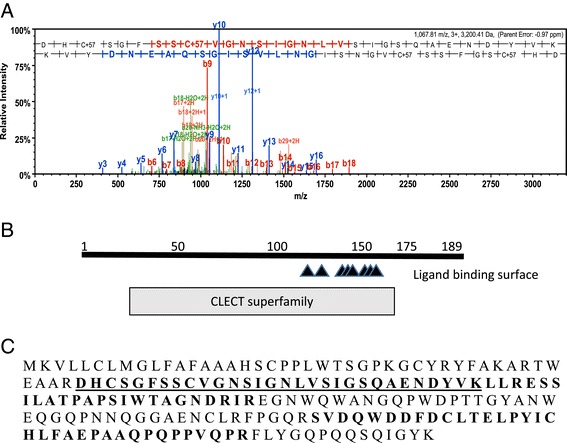
**Identification of a 21 kd protein with a C-type lectin domain. A. **Example spectra identifying Contig 1464 as encoding a protein occluded in the O. wendtii skeleton. The predicted domain structure is shown in **B. C: **The open reading frame of Contig 1464 with amino acid sequences identified using MS/MS in bold. The amino acids identified in the spectrum in **A** are underlined.

Figure 3 shows the sequence of SM24 as well as an example of a spectra showing the amino acid sequence of a peptide that matches the SM24 sequence. The sequence of the other peptides that matched are also shown. The low complexity domain is found at the carboxyl end of the protein and is enriched in proline, glutamine and asparagine that comprise a repetitive motif similar to what is found at the carboxyl end of the SM50 gene in euechinoids [7,14]. This includes a direct repeat of PQNPNQPG, which is similar but not identical to the SM50 repeat. Figure 4 shows a similar analysis of the SM21 gene, which also contains the C-type lectin domain and a low complexity region at the carboxyl end. This low complexity domain is also rich in proline and glutamine but lacks any direct repeats.

**Figure 5:**
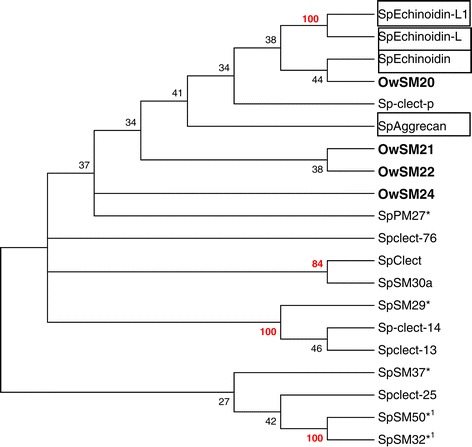
**A Maximum Likelihood tree showing the relationships among** ***O. wendtii*** **and** ***S. purpuratus*** **spicule matrix proteins found in the skeletal proteomes or identified through BLAST searches of the** ***S. purpuratus*** **genome using** ***O. wendtii*** **proteins as query.** Alignments are shown in Additional file 6 with Gblocks identified. *O. wendtii* proteins are in bold. Boxed proteins are those not found in the proteome; all others are found in skeletal proteomes. Proteins with *are those that had expanded repeats removed prior to alignment. 1 indicates two splice variants of the same gene. Bootstrap values of 50 or greater are showhn in red. Nodes with bootstrap values below 25 were collapsed. The accession numbers for the sequences used are as follows: [Genbank: XP_796721.1, NP_999775.1, NP_999630.1, NP_999804.1, XP_003726194.1, NP_999803.1, NP_999776.1, XP_781636.1, KJ999723, KJ999724, KJ999725, KJ999726, KJ999727].

We aligned the four *O. wendtii* C-type lectin proteins with all of the C-type lectin spicule matrix proteins from *S. purpuratus* and analyzed the relationships between the genes using the maximum likelihood algorithm in MEGA6 [20]. The *S. purpuratus* C-type lectins used in the analysis that are not found in the skeletal proteome were identified as top hits of BLAST queries of the *S. purpuratus* genome with the *O. wendtii* proteins. The most significant results indicate that the major spicule matrix proteins in *S. purpuratus* have diverged considerably from the *O. wendtii* proteins (Figure 5). Although the bootstrap values are low, it appears that the *O. wendtii* proteins are more similar to each other and to non-skeletal C-type lectins of *S. purpuratus* than they are to the other *S. purpuratus* spicule matrix proteins. SpPM27 shows some similarity to the *O. wendtii* proteins, but the data are not strong enough to assert common descent. The results suggest either that the co-option of most C-type lectin genes into playing a role in biomineralization in the two groups of echinoderms were two separate events that occurred some time after multiple C-type lectin genes were present in echinoderms, or that considerable diversification of the C-type lectin genes involved in biomineralization has occurred after genes became involved in that process in a common echinoderm ancestor. Clearly the extensive gene duplication and concerted evolution of repetitive regions seen in euechinoid spicule matrix genes has not occurred in O. wendtii, indicating it is specific to sea urchins. The repetitive, low complexity amino acid domains characteristic of sea urchin spicule matrix genes likely contributed to a rapid evolution of these gene’s sequences. While there are sequences in the *O. wendtii* C-type lectin proteins that are similar to the sea urchin base repeat unit (Figure 3), these apparently did not reach a repeat threshold that would lead to concerted evolution.

### A prevalent protein with unique domains

**Figure 6:**
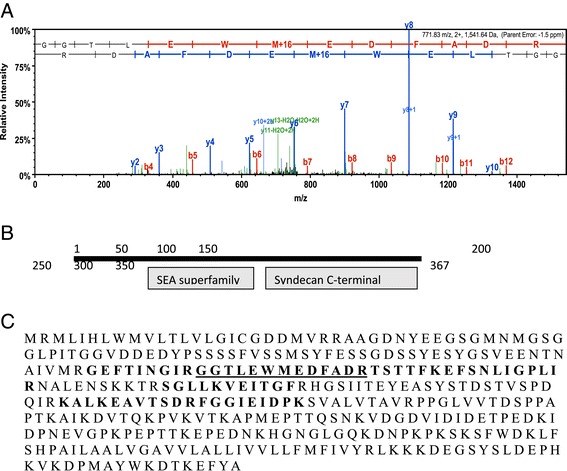
**Identification of a protein with a novel domain structure. A. **Example spectra identifying Contig 358 as encoding a protein occluded in the O. wendtii skeleton. The predicted domain structure is shown in **B. C: **The open reading frame of Contig 358 with amino acid sequences identified using MS/MS in bold. The amino acids identified in the spectrum in **A** are underlined.

Contig 00358 encodes an abundant protein in the brittle star skeletal proteome. When used as a query against the total NCBI database and the echinoderm database, two unique domains were identified within the protein. There was a well-conserved c-terminal syndecan domain that appears to be homologous to sea urchin syndecan [21], but there was also a less conserved SEA domain (sea urchin sperm, enterokinase, agrin domain) and several serine and acidic amino acid rich repetitive regions (Figure 6). Sea urchin syndecan is expressed as a minor component in the sea urchin spicule proteome. However only the C-terminal end of syndecan, which contains the internal and transmembrane regions, is conserved in the brittle star protein. Contig 00358 is a longer protein than the syndecan found in the sea urchin proteome and contains both the syndecan domain and an SEA domain. The SEA domain is not found in the sea urchin skeletal proteome and this combination of domains appears to be novel. Generally, SEA domains are involved in proteolysis and regulation of glycoproteins, which are functions consistent with a role in biomineralization.

### Proteins with Calcium binding domains

Among the other proteins detected in the O. wendtii skeletal proteome are several that have calcium binding domains as well as other domains that may play a role in mineralization (Tables 1 and 2). These include two proteins with high similarity to Ficolin, a lectin domain containing protein, which binds carbohydrates in a calcium dependent manner. Ficolin-like proteins have no previously described role in biomineralization. However, sea urchin fibrinogen related domain-containing protein (spFReD, XP_786278) is present in the sea urchin skeletal proteome [10-12]. Only a short sequence of this *O.* wendtii protein is known, but the known sequence does have a high level of sequence identity with SP-FReD and may be homologous. Calmodulin was found in both the O. wendtii and S. purpuratus skeletal proteome, as were two LDLreceptor-like protein with multiple calcium binding domains. LDL-Receptor-like proteins have been implicated as important in vertebrate bone formation [22].

### Proteases and protease inhibitors

We identified two matrix metalloproteases (MMPs) in the *O. wendtii* skeletal proteome. These are similar to those found in the sea urchin skeleton. We also found proteins in the O. wendtii proteome that contained kazal-type protease inhibitor domains. These domains are found in the SPARC and SPARC-like genes that gave rise to skeletal matrix proteins in vertebrates [4]. Sea urchins have genes related to SPARC in their genomes, but these proteins are not found in the skeletal proteome and have not been implicated in biomineralization in sea urchins [13]. One of the O. wendtii proteins with kazal inhibitor domains (Sequence ID 998, Table 1) was similar to agrin. Agrin is a glycoprotein that has a known role in the embryonic development of the neuromuscular junction, but is also thought to have additional roles. There are two agrin-like proteins that are found in sea urchin skeletal proteome, agrin-like; KAZAL-type inhibitor and agrin-like (XP_791472.3); Kazal serine proteinase inhibitor-like (XP_001181022.2). Protein 998 was most similar to the former. We also identified an alpha-2 macroglobulin-like protein similar to those found in *S. purpuratus* mineralized tissue (Table 1).

### MSP-130

One of the most prevalent proteins in the *S. purpuratus* skeletal proteome is MSP130. [10-12]. Recently evidence has been presented that MSP130 was introduced into the echinoderm clade by horizontal gene transfer [23]. The presence of this gene in all sea urchins [23] indicates that transfer occurred at least 250 MYA. MSP130 genes are also found in hemichordates, the sister group to echinoderms, suggesting the transfer occurred earlier. However, previous analysis of a brittle star gastrula transcriptome did not detect a MSP130 homologue [15]. Our analysis of the *O. wendtii* skeletal proteome also failed to detect MSP130 proteins. This suggests that the transfer of MSP130 occurred independently in sea urchins and hemichordates, or that the gene was lost in brittle stars.

### Miscellaneous proteins

A number of proteins identified in this study that are also found in the sea urchin skeletal proteomes may appear to be unrelated to mineralization. However, proteins similar to a number of them have also been identified as being part of the vertebrate bone and matrix vesicle proteomes [24-26]. This includes the LDL-receptors, gelsolin, calmodulin, alpha-2 macroglobulin, the cytoskeletal proteins, scavenger receptor, various ion channels and ubiquitin. Ubiquitin has recently been shown to have a functional role in biomineralization [27].

## Conclusions

This research represents the first direct description of echinoderm skeletal proteins outside of the sea urchin class. The enumeration and characterization of brittle star skeletal proteins presented here has also led to the identification of a number of interesting transcripts that likely correspond to biomineralization related proteins. These include C-type lectin domain containing proteins and a novel protein, corresponding to the transcript contig 00358. The presence of C-type lectin domain containing proteins in the mineral matrix was expected to some extent, but the identification of these proteins does have interesting evolutionary implications. While the use of transcriptome databases rather than a complete genome could have limited identification of all of the proteins in the brittle star skeletal proteome, this partial proteome has allowed us to identify a number of conserved proteins and domains.

Of the 38 identified proteins that were expressed in the brittle star skeletal proteome, most were also expressed in the sea urchin skeletal proteome. The presence of a similar complement of proteins occluded within the brittle star matrix indicates commonalities between brittle star and sea urchin and the similarity of cellular processes leading to biomineralization. However, the evolutionary origin of the C-type lectin domain containing biomineralization related proteins remains unknown. These proteins are critical to sea urchin biomineralization, but are also under unique evolutionary pressures that allow for rapid diversification of primary sequence. Additionally, their primary structure is such that it is difficult to identify homologs with primary sequence alone, which is largely the reason for using a proteomics approach to identify biomineralization related proteins. Four C-type lectin domain-containing proteins were identified by this technique, but none of these proteins had an obvious homolog in sea urchins. This information leaves open two possibilities regarding the evolution of this protein type in echinoderms. The proteins could have been shared in a brittle star/sea urchin ancestor and diverged in such a way that they are no longer recognizable between classes, or the proteins could have been co-opted independently in each class.

## Methods

### Skeletal preparation

Isolation of clean skeletal elements was modified from Mann et al. [10]. Arms of 2–8 adult brittle stars were removed close to the central disc, weighed, and rinsed under a jet of DI water. Rinsed arms were stirred vigorously for 2 hours in cold sodium hypochlorite (5% active chlorine, Acros Organics), with solution changes at 30-minute intervals. Enough bleach was used to comfortably cover the arms; approximately 10 ml bleach solution was used per gram of arms. Bleach incubation removed most organic material, including inter-vertebral cartilaginous tissue, resulting in partially ground mineral. The resulting vertebral ossicles and spines were washed 5× in cold, DI water, then ground with a mortar and pestle. Ground mineral was homogenized with homogenization buffer (4.5 M Guanidine Isothiocyanate, .05 M Sodium Citrate, 5% [v/v] β-mercaptoethanol, .5% (w/v) Sarkosyl) in a 40 mL homogenizer until well mixed and finely ground. Ground, homogenized skeletal elements were transferred to sterile 50 mL conical tubes and washed 5× in cold, sterile DI water, then incubated in cold sodium hypochlorite for one hour. After this incubation, skeletal elements were again washed 5× in cold, sterile DI water. This treatment resulted in finely ground mineral, free of attached organic material.

### Skeletal protein preparation

Purified skeletal elements were de-mineralized in cold acetic acid (~40 mL 25% (v/v)/g mineral) under gentle agitation until mineral was dissolved, generally 4–6 hours. After demineralization, the resulting solution had no mineral, but was slightly turbid. The precipitate and supernatant were transferred together to Spectra/Por 6 Dialysis Membrane MWCO 6-8000[Spectrum Laboratories, Greensboro, NC] for dialysis against a series of ~50× volume of TRIS base solutions. Dialysis solution was changed after 4,8, and 12 hours and a total dialysis time of 16 hours was allowed. The first round dialysis buffer was a molar excess of tris base calculated based on the amount of acetic acid used, followed by .01 M, .001 M, and .0001 M tris base for the final rounds of dialysis. After dialysis, a small amount of precipitate remained, but was removed before further processing. This insoluble precipitate was largely insoluble in sample buffer and did not result in significant banding when separated by SDS-PAGE (data not shown). The soluble fraction was concentrated using Amicon Ultra-15 centrifugal filter units, followed by Amicon Ultra-0.5 centrifugal filter units (3000 NMWL)(Millipore, Bellerica, MA). After centrifugal filtration, the retentate was concentrated approximately 50×.

### Liquid sample preparation

Samples were concentrated by buffer exchange against HPLC grade water using a 5 kDa molecular weight cut-off filter. The sample was processed by solution digestion using standard protocols. The samples were reduced with 8 mM dithiothreitol at 60°C followed by alkylation with 10 mM iodoacetamide at RT and digested with sequencing grade trypsin (Promega, Madison, WI) at 37°C for 4 h. Digested samples were quenched with formic acid and the supernatant was analyzed directly without further processing.

### Gel fractioned sample preparation

Protein quantitation was performed by Qubit Fluorometry (Invitrogen, Carlsbad, CA). 20 μg of material was separated on a 4-12% Bis Tris NuPage gel (Invitrogen, Carlsbad, CA) in the MOPS buffer system. In one experiment the gel lane was excised into twenty equally sized segments. In another, the three major bands were excised and analyzed. Using a robot (ProGest, DigiLab), gel pieces were reduced with 8 mM dithiothreitol at 60°C followed by alkylation with 10 mM iodoacetamide at RT. Samples were then digested with sequencing grade trypsin (Promega, Madison, WI) at 37°C for 4 h and quenched with formic acid. The supernatant was analyzed directly without further processing.

### Mass spectrometry

For each sample [from 3 excised gel slices, 1 unfractioned liquid sample, and 20 fractioned gel slices], LC-MS/MS was performed identically by MS Bioworks (Ann Arbor, Michigan). The digested sample was analyzed by nano LC/MS/MS with a Waters NanoAcquity HPLC system interfaced to a ThermoFisher LTQ Orbitrap Velos. Peptides were loaded on a trapping column and eluted over a 75 μm analytical column at 350 nL/min; both columns were packed with Jupiter Proteo resin (Phenomenex). The mass spectrometer was operated in data-dependent mode, with MS performed in the Orbitrap at 60,000 FWHM resolution and MS/MS performed in the LTQ. The fifteen most abundant ions were selected for MS/MS.

### Data processing

Data were searched using a local copy of Mascot against the NCBI non-redundant protein database as well as the CSULB All Contigs and Singletons database (454-sequenced brittle star gastrula stage transcriptome) and a tube foot illumina cDNA database. The Contigs and Singletons database was also translated in all six frames using Proteomatic 1.2.1 (University of Munster) to visualize reading frame. The translated database was appended with common contaminants, reversed and concatenated. Trypsin was input as the digestive enzyme, mass values were monoisotopic, the peptide mass tolerance was 10 ppm, the fragment mass tolerance was 0.8 Da, and the maximum number of missed cleavages was 2. Carbamidomethylation (C) was set as a fixed modification and variable modifications included oxidation (M), acetyl (N-term), pyro-glu (N-term Q), and deamidation (N,Q). Mascot DAT files were parsed into the Scaffold software for validation, filtering and to create a non-redundant list per sample. The data were filtered using a minimum protein value of 90%, a minimum peptide value of 50% (Prophet scores) and requiring at least two unique peptides per protein.

### PCR and sequencing of full length cDNAs

Selected proteins identified through comparison to transcriptome sequences that did not contain the full length coding sequence were analyzed further. mRNA from gastrula stage embryos was converted to cDNA and used in 5’ and/or 3’ RACE reactions to obtain full length sequences following the manufacturers protocol (Ambion, Austin, TX). Products were cloned into TA vectors (Invitrogen, Carlsbad, CA) and sequenced by Macrogen [Rockville, MD] using Sanger sequencing methods.

### Phylogenetic analysis

Expanded repeats in spicule matrix proteins were removed. The Protein sequences were then first aligned in CLUSTAL X. They were then imported into MEGA6 [20]. Gblocks [28] was used to identify conserved regions, and sections of the proteins were realigned in MEGA6 keeping these regions as anchors. A maximum likelihood tree with 1000 bootstraps was constructed in MEGA6. Various methods of phylogenetic analysis were tested, and while there were some variations in groupings the relationships between the proteins from the two species was consistent.

## Additional files

Additional file 1:
**Spicule matrix proteins from 20 SDS PAGE gel slices compared to gastrula transcriptome identified with >90% confidence.** The files lists gastrula transcript ID, proteins identified, Protein ID probability, number of unique peptide counts, unique spectrum counts, total spectrum counts, peptide sequence, best peptide ID probability, best Mascot score and best Mascot identity score. Additional file 2:
**Spicule matrix proteins from total unfractionated sample compared to gastrula transcriptome identified with >90% confidence.** The files lists gastrula transcript ID, proteins identified, Protein ID probability, number of unique peptide counts, unique spectrum counts, total spectrum counts, peptide sequence, best peptide ID probability, best Mascot score and best Mascot identity score. Additional file 3:
**A. Example spectra identifying transcript LUPK as encoding a protein occluded in the O. wendtii skeleton.** The predicted domain structure is shown in B. C: The open reading frame of LUPK with amino acid squences identified using MS/MS in bold. The amino acids identified in the spectrum in A are underlined. Additional file 4:
**Spicule matrix protein from 3 major SDS PAGE bands compared to gastrula transcriptome identified with >90% confidence.** The files lists gastrula transcript ID, proteins identified, Protein ID probability, number of unique peptide counts, unique spectrum counts, total spectrum counts, peptide sequence, best peptide ID probability, best Mascot score and best Mascot identity score. Additional file 5:
**Spicule matrix proteins from 20 SDS PAGE gel slices compared to tube foot transcriptome identified with >90% confidence.** The files lists gastrula transcript ID, proteins identified, Protein ID probability, number of unique peptide counts, unique spectrum counts, total spectrum counts, peptide sequence, best peptide ID probability, best Mascot score and best Mascot identity score. Additional file 6:
**Alignment of C type lectins from** ***O wendtii*** **and** ***S purpuratus*** **skeletal proteomes.** This file shows a MEGA6 alignment of the sequences used to construct the tree shown in Figure 5. Conserved regions identified with Gblocks are indicated.

## Abbreviations

*O. wendtii*: *Ophiocoma wendtii*
*S. purpuratus*: *Strongylocentrotus purpuratus*
SDS-PAGE: Sodium dodecyl sulfate polyacrylamide gel electrophoresis
HPLC: High pressure liquid chromatography
LC/MS/MS: Liquid chromatography/mass spectrometry/mass spectrometry
