# Supplementary material for: Examination of the skeletal proteome of the brittle star Ophiocoma wendtii reveals overall conservation of proteins but variation in spicule matrix proteins
Source: Proteome Sci. 2015 Feb 7;13:7. doi: 10.1186/s12953-015-0064-7 (PMC4336488; doi:10.1186/s12953-015-0064-7)
Supplement: Additional file 3: — A. Example spectra identifying transcript LUPK as encoding a protein occluded in the O. wendtii skeleton. The predicted domain structure is shown in B. C: The open reading frame of LUPK with amino acid squences identified using MS/MS in bold. The amino acids identified in the spectrum in A are underlined. [file 12953_2015_64_MOESM3_ESM.pdf]

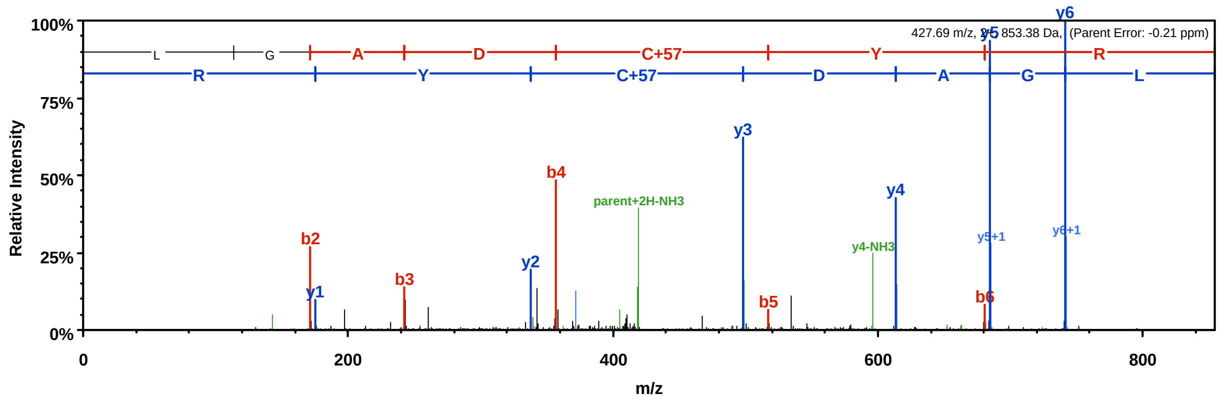

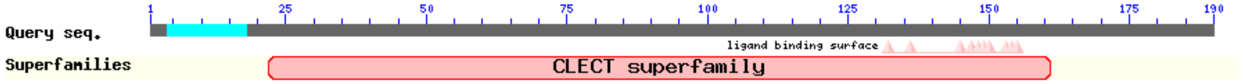

M K I L S L L V A A G L L A L A N A H *G G C P P L W T K* ***<u>L G A D C Y R</u>*** ***Y F G F P R P M R***
I A E R V C A Q F T S C S D A G T T A V A H L A S I A N G A Q N T Y L Y N L I E S V A P E L P
P A P V W I G L N D I V **R E G D F R W P D G T Q Y V F K N W E R G H P N N Q G N S**
D C V A M P G E R P G A G W V S M N C M E E F A Y I C K M P A D V S Q E V P A Q P A
P Y Y W D S Y P E L Y T N Y Y Y

Additional file: SM 22 sequence. A. Example spectra identifying LUPK as encoding a protein occluded in the O. wendtii skeleton. B. The predicted domain structure. C: The open reading frame of LUPK with amino acid squences identified using MS/MS in bold. The amino acid sequences deduced from a short transcript that contain the original peptide are italicized. The original peptide is shown in the spectra and underlined. All other peptide matches shown in bold were found after obtaining the complete coding sequence and re-anlaysis with the peptide data.
