# Supplementary material for: Examination of the skeletal proteome of the brittle star Ophiocoma wendtii reveals overall conservation of proteins but variation in spicule matrix proteins
Source: Proteome Sci. 2015 Feb 7;13:7. doi: 10.1186/s12953-015-0064-7 (PMC4336488; doi:10.1186/s12953-015-0064-7)
Supplement: Additional file 6: — Alignment of C type lectins from O wendtii and S purpuratus skeletal proteomes. This file shows a MEGA6 alignment of the sequences used to construct the tree shown in Figure 5. Conserved regions identified with Gblocks are indicated. [file 12953_2015_64_MOESM6_ESM.pdf]

Gblocks 0.91b Results

Processed file: **ClectinNov18.v2.fas**
Number of sequences: **20**
Alignment assumed to be: **Protein**
New number of positions: **55** (selected positions are underlined in blue)

```
                            10        20        30        40        50        60
                    =========+=========+=========+=========+=========+=========+
SpEchinoidin-L1     CPPLWTAFQNNCYRYFSVKNITWLGAEMHCSGFSVPCSDVDSTINLGHLTSIHSKEKMTF
SpEchinoidin-L      CPPLWTAFQNNCYRYFSVKNITWLGAEMHCSGFSVPCSDVDSTISLGHLTSIHSKEEMTF
SpEchinoidin        CPKLWTGFRNHCYRFF-SHELTWLAAENFCRSFTVPSWGEVTRTNSGHLVSIHSQPEQDF
OwSM20              CRPLWTRFQNNCYRFF-GEATTWKEAESSCRQFV-TV------SKPGHLVSIHSKDENDF
Sp-clect-p          CPTFWTGYGNYCYRYF-NDVKTWLEAEFYCRTFG-AG-GCMGQTGQAHLVSIHSQDENDF
OwSM21              CPPLWTSGPKGCYRYF-AKARTWEAARDHCSGFS-SC----VGNSIGNLVSIGSQAENDY
OwSM22              CPPLWTKLGADCYRYF-GFPRPMRIAERVCAQFT-SC-SDAGTTAVAHLASIANGAQNTY
OwSM24              CPPLWTHLGDSCYRFF-PDRLRWEDAKTTCGQFA-PC----SAEALGQLVKIADAQVNEF
Sp-clect-14         CIPGWTNFGKYCYKFF-NQRMNWLQAQRECNKYS-AL---NGTTPRGYLMTSKDQQHNNF
Spclect-13          CQPGWTNFGKFCYKYF-YERLPFMEAQMQCLSFR-AS----PLAPRGYLITAKDQTHNRF
SpSM29              CLPGWTNFGNFCYKYI-WQRLPYDEANMFCARFS-AQRPGFGMPPRGHLIVTKDMMHNKF
SpSM50              ------------------MRVPYRMASEFCEMVT-PC--GNGPAKMGALASVSSPQENME
SpSM32              CPAYYSQSGQSCYRYF-NIPLAYQWASEFCEMVT-PC--GNGPAVMGTLAAPKSPQENME
SpAggrecan          CPHAWTQRNGYCYRYF-DDMLPWSVAESRCHEHF-DS------DGIAHLVSIHDQAENDF
Spclect-25          CPFGWSQSGRSCYKVS-QMAATYEQAAMLCSQQS-SC----ETGFGGHLVSITTDFEKQQ
SpPM27              CKGGWFLIGQQCFKMM-SRALKWNDAELMCEQNA-PC----GTPVLGGVMTIPDIQTSNA
SpClect             CAPYFTYNNGSCYRMSRLEGMPFAQANLFCGQCH-PG---------AHLVVISDAAEHEF
SpSM30a             CAKFWVQEGNSCYLFDNENGLFQAAANMYCGQMH-PN---------ASLVTVNSLAENNF
SpSM37              CPPSWVKVENSCYRAF-GQLKTYDMASQSCKTFT-GC----DGISPGHLAAPTTFEERRA
Spclect-76          --------------------MTFNEATFFCNRYG------------GSLYALDSPSKNRI

                            70        80        90       100       110       120
                    =========+=========+=========+=========+=========+=========+
SpEchinoidin-L1     LSGLHESLRSKLVTSTT-------------YVWIGLHDQTTEDSWEWSDGSSLD------
SpEchinoidin-L      LSVLYESIRSKVVTSTT-------------YVWIGLHDKTTEASWEWSDGSSQD------
SpEchinoidin        VTALYESSRKKGDENA--------------GHWFGLHDTSTDGTFEWTDGTPVD------
OwSM20              V---YTLWQSHLISGLRDSDYPGVVDRTN-AVLIGLNDLATEGTYAWSDGTLVD------
Sp-clect-p          L---FSYFDTVRNKLPPAIGPIRDE-----RLWLGLTDKKQEGFWTWSDGTNLD------
OwSM21              V----KLLRESSILATPAP-----------SIWTAGNDRIREGNWQWANGQPWD--PTTG
OwSM22              L---YNLIESVAPELPPA------------PVWIGLNDIVREGDFRWPDGTQYV------
OwSM24              L---GIYLNSFVPQPPAT------------DFWIGASDLGLEGRFTNVDGTPVT------
Sp-clect-14         Q---HNWLRYTGGGLN--------------KVWLGLSEKTNN-NYFWADGTPLL---PTQ
Spclect-13          Q---HNFLTFTGGANN--------------KVWMGLAERQYNVFW-WADGTPFR---PHD
SpSM29              Q---HNWLRYTGGGAN--------------KIWMGLAELPSAPRYFWADGTEFFFTRNFV
SpSM50              I---YQLVAGFSQDNQMEN-----------EVWLGWNSQS---PFFWEDGTPAYPNGFAA
SpSM32              I---YRLVASFSQDNQMER-----------EVWLGWNSMN---PFMWENGAPAYPHGFSA
SpAggrecan          I---FDIFNSANGDHGDLDITNTPGTDLPTGMWTGLHQEVRDGPWVWSDGSSND------
Spclect-25          V---TDILNFNQMARN--------------ETWLGL--RTYNGPLRWASGEPLN----PA
SpPM27              V---INHLKSLSSTAMAIDI----------PFWTGLHNKRYEG-WKWPAGQPLR------
SpClect             L---YNNFYGFEPT----------------EVWVGLFFNSTTLAWEWVNGEPA-------
SpSM30a             L---YEWAVRMMVEPE--------------PVWIGLHA-GPMGQWQWYSGEPVT------
SpSM37              VKIRFDLLRPIGGNRIGEGQRLRPS-----NVWMGFR-LGQGANSSWDSTEQYD---NLL
Spclect-76          M---LSYLTTVFGQGRRQ------------KFWTGFYKAENALGWRWSTGSTSQ------
```

```
                           130       140       150       160       170       180
                  =========+=========+=========+=========+=========+=========+
SpEchinoidin-L1   Y-EIWEP--GQPSSHN---G--NQDCVMFFSS------------------NKYKWNDLAC
SpEchinoidin-L    Y-EIWAS--GQPNNYG---G--NQDCGVFSST------------------SEYKWNDFAC
SpEchinoidin      F-THWKP--GQPDNYPNHVG--NENCGVMRSH------------------SGSQWKDGRC
OwSM20            FGPYWKP--DQPNNYG---G--NQDCSSMHKRDG----------------TDDGWNDIVC
Sp-clect-p        Y-ANWRA--GEPNNNG---G--NEDYAQMSVEIN----------------HEGLWFDMTD
OwSM21            Y-ANWEQ--GQPNNQG---G--AENCLRFPGQR-----------------SVDQWDDFDC
OwSM22            F-KNWER--GHPNNQG------NSDCVAMPGER-----------------PGAGWVSMNC
OwSM24            F-TNWAQ--GQPDNGAQSTGQ-PENCVMYGG-------------------ETKLWSDASC
Sp-clect-14       W-NKWKI--DQPQLNA------HIQGVHTFDNMV-----------------DMTWVTSFY
Spclect-13        W-NLFKP--DQPQQNH------HIDGVFTFDNY-----------------PYQTWVTSSH
SpSM29            F-NRFKP--DQPQQNA------HRQAVHSFNNR-----------------PDNSWVTTSV
SpSM50            FSSSWPVNPQNPMSGP------PGRAPVMKRQNPPVRPGQGGRQIPQG--VGPQWEAVEV
SpSM32            FDSGWPVNTRNPFGMP------PGFAPVMRRELGTIPGRQGPNRRMIPASQGPVWQVAEL
SpAggrecan        Y-THWLP--GQPDNGYHARGRLNEDCVIIWRNDDNGSE------------STRQWDDIGC
Spclect-25        LTNPWHP--GNPMRG-------PNMCTVS---------------------FQNRWATRNC
SpPM27            F-VNWAP--REPNNQLLDQQ--HSYCARMN--------------------RMGQWYVVRC
SpClect           LNVPWEPFYGEPLVTH------QGAAMMYVSEVIDQQTTLKYG-------YGIEVSDYLV
SpSM30a           Y-TNWERM-TAPMAEP------GLGAMIFDADIIAQMFNNQVE-------ITPQWVPEQA
SpSM37            LDQHWGP--NRPNMPGQRPQAQAFDCMTLPGQMQMNG-------------AVQKWGHIAC
Spclect-76        Y-TDWEA--GKPATTN------EAIGAVAWG-------------------GTHHWSDTWQ

                           190
                  =========+=======
SpEchinoidin-L1   DSDGDTAYHGSAYVCKL
SpEchinoidin-L    DSDGDSPHHATAYVCKL
SpEchinoidin      VQPSDSAIHY--FICKT
OwSM20            GAGDEGPLP---YICKI
Sp-clect-p        AHGDYIVH----YICKM
OwSM21            LTELP-------YICHL
OwSM22            MEEFA-------YICKM
OwSM24            AMMMP-------YICMM
Sp-clect-14       KTEMS-------FICQY
Spclect-13        ETQMS-------FTCQY
SpSM29            ETEVS-------FICQY
SpSM50            TAMRA-------FVCEV
SpSM32            TGPTHA------FVCEV
SpAggrecan        DIQGGMP-----YVCKM
Spclect-25        NMRRQ-------FICEM
SpPM27            DEPMY-------FACSM
SpClect           SELGEEPLP---FICEY
SpSM30a           INDRHA------LICEY
SpSM37            NMQAA-------YMCEI
Spclect-76        DEPLSASFA---FICQA
```
